# Seasonality of Human Leptospirosis in Reunion Island (Indian Ocean) and Its Association with Meteorological Data

**DOI:** 10.1371/journal.pone.0020377

**Published:** 2011-05-31

**Authors:** Amélie Desvars, Sylvaine Jégo, Frédéric Chiroleu, Pascale Bourhy, Eric Cardinale, Alain Michault

**Affiliations:** 1 Unité Mixte de Recherche Contrôle des Maladies Animales Exotiques et Emergentes (UMR CMAEE), Centre de Coopération Internationale en Recherche Agronomique pour le Développement (CIRAD), Sainte-Clotilde, La Réunion, France; 2 Centre Régional de Recherche et de Veille sur les Maladies Emergentes de l'Océan Indien (CRVOI), Sainte-Clotilde, La Réunion, France; 3 UMR Peuplement Végétaux et Bioagresseurs en Milieu Tropical (PVBMT), CIRAD, Saint-Pierre, La Réunion, France; 4 Institut Pasteur, Centre National de Référence des Leptospiroses, Unité Postulante de Biologie des Spirochètes, Paris, France; 5 Laboratoire de Bactériologie-Parasitologie-Virologie-Hygiène, Groupe Hospitalier Sud Réunion (GHSR), Centre Hospitalier de La Réunion (CHR), Saint-Pierre, La Réunion, France; University of California Merced, United States of America

## Abstract

**Background:**

Leptospirosis is a disease which occurs worldwide but particularly affects tropical areas. Transmission of the disease is dependent on its excretion by reservoir animals and the presence of moist environment which allows the survival of the bacteria.

**Methods and Findings:**

A retrospective study was undertaken to describe seasonal patterns of human leptospirosis cases reported by the Centre National de Références des Leptospiroses (CNRL, Pasteur Institute, Paris) between 1998 and 2008, to determine if there was an association between the occurrence of diagnosed cases and rainfall, temperature and global solar radiation (GSR). Meteorological data were recorded in the town of Saint-Benoît (Météo France “Beaufonds-Miria” station), located on the windward (East) coast. Time-series analysis was used to identify the variables that best described and predicted the occurrence of cases of leptospirosis on the island. Six hundred and thirteen cases were reported during the 11-year study period, and 359 cases (58.56%) were diagnosed between February and May. A significant correlation was identified between the number of cases in a given month and the associated cumulated rainfall as well as the mean monthly temperature recorded 2 months prior to diagnosis (*r* = 0.28 and *r* = 0.23 respectively). The predictive model includes the number of cases of leptospirosis recorded 1 month prior to diagnosis (*b* = 0.193), the cumulated monthly rainfall recorded 2 months prior to diagnosis (*b* = 0.145), the average monthly temperature recorded 0 month prior to diagnosis (*b* = 3.836), and the average monthly GSR recorded 0 month prior to diagnosis (*b* = −1.293).

**Conclusions:**

Leptospirosis has a seasonal distribution in Reunion Island. Meteorological data can be used to predict the occurrence of the disease and our statistical model can help to implement seasonal prevention measures.

## Introduction

With more than 500,000 cases per year, leptospirosis is one of the most widespread diseases in the world [1]. *Leptospira* spp. (phylum Spirochaetes) are bacteria composed of both saprophytic and pathogenic members, such as *Leptospira biflexa* sensu lato and *Leptospira interrogans* sensu lato, respectively [2]. Leptospires are motile, aerobic and slow-growing bacteria that have an optimal growth temperature of 30°C and are able to survive in soil and water for long periods [2]. Although asymptomatic infections are common in humans, the disease can also be lethal [3]. The incidence is significantly higher in tropical countries than in temperate regions, mainly because of the longer survival period of leptospires in a warm, humid environment [4]. Infection in humans is either via direct contact with the urine of an infected animal or indirectly via the contaminated environment [4]. The latter is the major source of leptospirosis infections in tropical areas [5]. Rodents are the most efficient epidemiological reservoirs for pathogenic leptospires [6], [7] but dogs are also a significant reservoir responsible for human infection in tropical countries [8], [9]. Many sporadic cases of leptospirosis in tropical regions occur following exposure during simple day-to-day activities [10], [11]. Many infections result from walking barefoot in damp conditions or gardening with bare hands [12]. Moreover, recent outbreaks such as the 1998 Lake Springfield Triathlon [13] and the 2000 Borneo Eco-Challenge [14] outbreaks revealed the risk of exposure to infection to leisure activities.

As the growth of *Leptospira* is very slow, the culture of blood or urine samples in a specific medium is rarely conducted for clinical diagnosis. The microscopic agglutination test (MAT), is the serological standard reference test for diagnosing leptospirosis. A positivity threshold of 1/100 is used in metropolitan France but, since 1999, the threshold of 1/400 has been used in high-risk French overseas territories such as Reunion Island [15]. Since 1997, DNA amplification has also been used in Reunion Island as a standard method of diagnosis [16]. Polymerase-chain reaction (PCR) with specific *Leptospira* primers allows the bacteria in patients' serum to be detected from the onset of symptoms to the appearance of antibodies, thus increasing the rate of early diagnosis in the island.

Diagnosis of leptospirosis is a reliable, standard procedure. Nevertheless, there is a need for a leptospirosis early warning system to predict when and where leptospirosis epidemics may occur. Unusual meteorological conditions, such as high rainfall, flooding or El Niño effects are often cited retrospectively as the precipitating factors for epidemics [17], [18], [19], [20] and might be useful in predicting the number of cases of leptospirosis.

In this study, we present leptospirosis cases in Reunion Island from 1998 to 2008. The objectives of this study were to describe seasonal patterns of leptospirosis reported in Reunion Island, and to determine if cases were associated with rainfall, temperature and global solar radiation (GSR). Results are discussed according to geographical, meteorological and socio-economical aspects. Our model showed that data on rainfall, temperatures and GSR could be successfully used to predict the number of cases in a given month. Consequently, if risk periods can be predicted, early steps can be taken to prevent infections, thus reducing the monthly number of cases.

## Materials and Methods

### Presentation of Reunion Island

**Figure 1:**
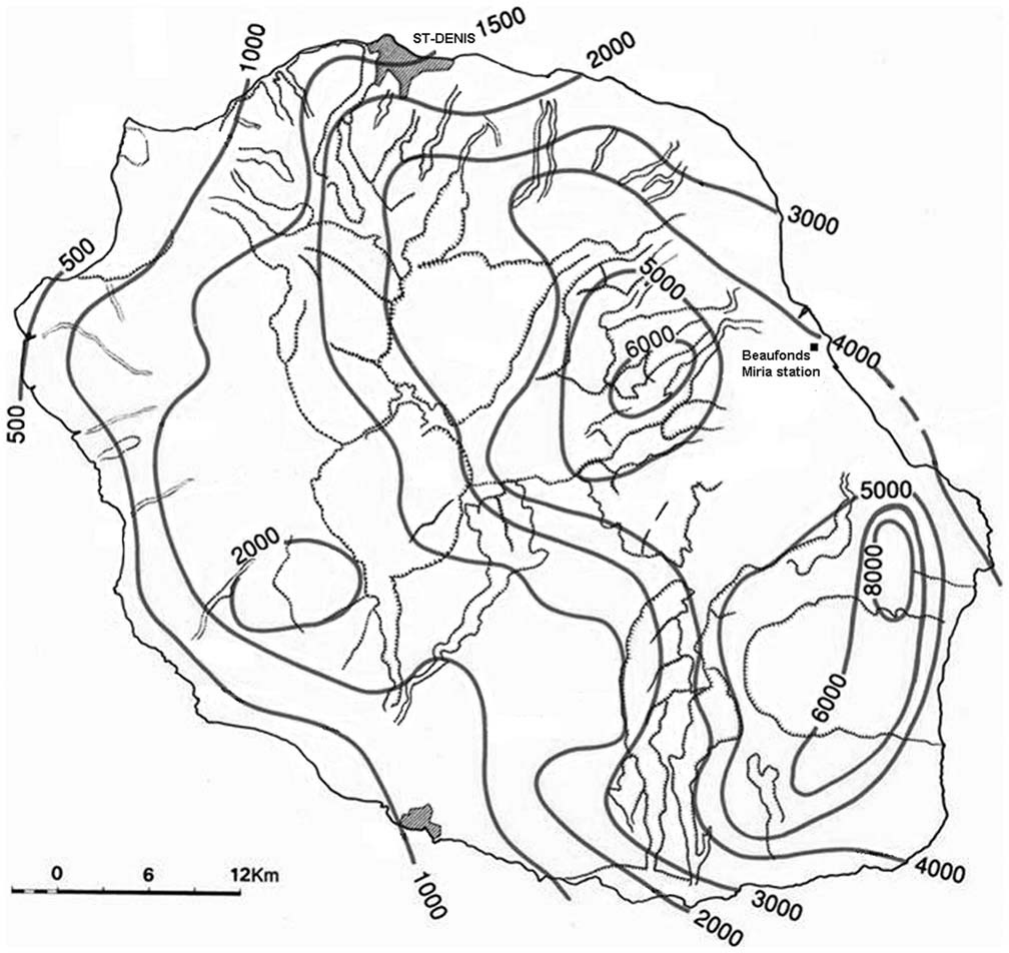
Annual rainfall in Reunion Island (from [**21**]). Isohyetal lines and location of the Météo France meteorological Beaufonds-Miria station.

The island is situated in the south-west Indian Ocean at 55°30′ East and 21°05′ South. It is 800 km East of Madagascar. The island is part of the Mascareignes archipelago and covers 2,510 km^2^ [21]. According to the 2006 census [22], Reunion Island has 781,962 inhabitants, with a population density of 312.3 people per km^2^. The population is concentrated in coastal towns, while the south-eastern area containing the Piton de la Fournaise volcano is sparsely populated [22]. The climate is tropical and temperatures are the highest from January to March, with a cool season between July and September. The eastern coast (or “windward” coast), has rainfall of around 2,000 mm per year, whereas the western coast (or “leeward” coast), has an annual rainfall of less than 2,000 mm (**Figure 1**) [21].

### Data collection

#### Human cases

Leptospirosis has been a notifiable disease in France since 1986, but data about the disease are centralized and diffused by the Centre National de Références des Leptospiroses (CNRL) of the Pasteur Institute (Paris). The monthly number of cases was found in the annual reports of the CNRL (http://www.pasteur.fr/recherche/Leptospira/LeptospiraF.html), which reports cases confirmed by at least one positive biological test (patients with a MAT titer ≥1/400, or with a positive PCR result, or with a positive blood culture). Based on voluntary participation, data from local hospitals are transmitted yearly to the CNRL. At the time of our analysis, serological data for Reunion Island were available for the period 1998–2009.

#### Climatic data

Meteorological data was obtained from the station of “Beaufonds-Miria” (run by Météo France, 21.0466°S.; 55.7266°E.) in Saint-Benoît, a town located on the eastern coast (altitude 7 m). We chose this station because the eastern coast presents the highest seroprevalence of the disease [23]. We collected the following data: minimum, maximum and average temperature in degrees Celsius; cumulated, average, minimum and maximum rainfall in millimetres and GSR (the incident radiation of short wavelength an horizontal area receives [21]) in Joules per square centimetre.

### Statistical analysis

Statistical analysis was conducted with the TSA package of the statistical software R (http://www.stat.uiowa.edu/~kchan/TSA.htm) [24]. Time-series analysis [25] was used to identify temporal patterns in the series of cases of leptospirosis, and the relationship between rainfall, temperature or GSR, and the diagnosis of leptospirosis in Reunion Island between 1998 and 2008. Time-series analysis is a method that allows inferences to be drawn from data consisting of serial observations which are correlated over time, by incorporating correlated error terms into the model that is used [25]. To stabilize the variance, a log transformation of each observation was made for further analysis. Autocorrelation (ACF) and partial autocorrelation (PACF) functions were calculated to represent seasonal and cyclical trends in time-series of cases of leptospirosis. After removing the seasonality of each series, the stationarity of each one was tested using ACF and PACF, and using the augmented Dickey-Fuller test. An Auto-Regressive Integrated Moving-Average with eXogeneous variables model, ARIMAX (*p*, *d*, *q*), was fitted to the time-series of cases of leptospirosis, with *p* the number of autoregressive parameters, *d* the number of differencing passes, and *q* the number of moving average parameters. The study of cross-correlation functions between the time-series of cases of leptospirosis and each meteorological time-series allowed the potential lags to be identified in the different models tested [25]. The best-fitting model was selected based on the goodness-of-fit criterion, Akaike's Information Criterion (AIC), and coefficients were tested using Student's test.

## Results

The 11-year data set consists of human cases of leptospirosis reported each month to the CNRL by hospitals in Reunion Island, and meteorological data from a station in Saint-Benoît collected between 1^st^ January 1998 and 31^st^ December 2008.

### Meteorological data

Annual and monthly meteorological data for the 11-year study period are summarized in **Tables 1** and **2** respectively. Cumulated rainfall, mean rainfall and maximum rainfall were highly correlated with minimum temperature, mean temperature and maximum temperature, >0.80 and >0.95 respectively (*data not shown*). Consequently, we chose to keep only the cumulated rainfall and mean temperature variables for further analysis.

**Table 1:** Summary of the annual meteorological data in Reunion Island.

Year	Cumulated annual rainfall, in mm	Maximum monthly rainfall, in mm (month)	Minimum monthly rainfall, in mm (month)	Average monthly rainfall, in mm	Mean monthly minimum temperature, in °C (± S.E.)	Average annual temperature, in °C (± S.E.)	Mean monthly maximum temperature, in °C (± S.E.)	Mean annual solar global radiation, in Joules/cm^2^ (± S.E.)
1998	3412	1520.4 (Feb)	62.6 (Mar)	11.47 (±5.07)	19.52 (±0.67)	23.02 (±0.64)	27.23 (±0.60)	1811.68 (±128.00)
1999	3039.8	916.6 (Mar)	27 (Oct)	8.41 (±2.36)	18.86 (±0.57)	22.67 (±0.55)	27.07 (±0.53)	1895.97 (±107.77)
2000	2714.8	594 (Feb)	41.4 (Sep)	7.51 (±1.64)	19.1 (±0.63)	23.73 (±0.62)	26.99 (±0.57)	1831.12 (±100.60)
2001	2145	480.6 (Jan)	17.6 (Sep)	6.7 (±1.76)	19.38 (±0.61)	23.07 (±0.62)	27.4 (±0.64)	1830.57 (±87.86)
2002	2780.8	410.6 (Mar)	72.6 (Oct)	8.22 (±1.17)	19.41 (±0.59)	23.05 (±0.61)	27.18 (±0.58)	1808.35 (±106.88)
2003	2752	643.2 (Apr)	59.2 (Oct)	8.71 (±1.77)	19.76 (±0.69)	23.43 (±0.64)	27.95 (±0.62)	1777.88 (±114.39)
2004	4465.2	966.2 (Dec)	86.6 (Oct)	12.4 (±2.29)	19.72 (±0.67)	23.25 (±0.66)	28.01 (±0.66)	1685.18 (±90.20)
2005	3679.2	826.2 (Feb)	76 (Jul)	10.45 (±2.46)	19.58 (±0.72)	23.3 (±0.70)	28.24 (±0.68)	1760.97 (±102.22)
2006	2795.2	629.8 (Feb)	78.4 (Oct)	8.08 (±1.72)	20.05 (±0.61)	23.44 (±0.62)	27.77 (±0.65)	1819.22 (±105.34)
2007	2981.8	782 (Jan)	37.4 (Nov)	8.27 (±2.31)	19.87 (±0.67)	23.44 (±0.64)	28.22 (±0.58)	1788.03 (±99.20)
2008	3434.4	632.2 (Jan)	82.2 (Apr)	9.68 (±1.63)	19.57 (±0.66)	23.38 (±0.66)	28.51 (±0.63)	1858.21 (±101.36)

Presentation of the yearly data on cumulated and maximum rainfall, minimum, maximum, and mean temperature, and mean global solar radiation, from 1 January 1998 to 31 December 2008 (Beaufonds-Miria station, Saint-Benoît).

**Table 2:** Summary of monthly meteorological data.

Month	Mean of monthly cumulated rainfall, in mm (± S.E.)	Mean rainfall, in mm (± S.E.)	Mean of maximum rainfall, in mm (± S.E.)	Mean of minimum temperature, in °C (± S.E.)	Average monthly temperature, in °C (± S.E.)	Mean of maximum temperature, in °C (± S.E.)	Mean of monthly global radiation in Joules/cm^2^ (± S.E.)
January	439.40 (±51.48)	14.66 (±1.60)	120.75 (±22.47)	22.15 (±0.21)	25.79 (±0.15)	30.10 (±0.20)	2091.78 (±61.03)
February	603.04 (±107.37)	23.35 (±4.69)	163.73 (±27.39)	22.49 (±0.22)	25.98 (±0.15)	30.30 (±0.23)	1905.64 (±78.57)
March	366.56 (±71.05)	12.88 (±2.20)	74.33 (±14.66)	21.95 (±0.18)	25.51 (±0.16)	30.00 (±0.24)	1892.59 (±36.62)
April	290.29 (±47.84)	10.48 (±1.82)	86.27 (±19.71)	20.81 (±0.23)	24.52 (±0.16)	29.24 (±0.24)	1711.15 (±48.98)
May	172.55 (±34.00)	6.06 (±1.22)	39.29 (±10.28)	19.36 (±0.26)	22.91 (±0.16)	27.55 (±0.17)	1391.38 (±46.34)
June	153.15 (±27.50)	5.20 (±0.93)	47.05 (±11.90)	17.27 (±0.17)	21.04 (±0.15)	25.90 (±0.19)	1336.56 (±11.34)
July	209.73 (±28.96)	7.05 (±0.92)	58.60 (±9.94)	16.71 (±0.19)	20.25 (±0.12)	24.79 (±0.16)	1346.75 (±27.95)
August	148.15 (±18.95)	4.94 (±0.59)	38.00 (±8.20)	16.85 (±0.15)	20.41 (±0.09)	25.00 (±0.22)	1572.64 (±23.44)
September	164.35 (±29.48)	5.62 (±1.01)	44.80 (±7.06)	17.47 (±0.17)	21.20 (±0.13)	25.77 (±0.21)	1910.19 (±42.99)
October	96.84 (±14.41)	3.20 (±0.45)	27.85 (±4.22)	18.29 (±0.15)	22.04 (±0.14)	26.52 (±0.20)	2114.38 (±43.97)
November	160.07 (±31.24)	5.37 (±1.04)	52.16 (±13.55)	19.71 (±0.16)	23.43 (±0.20)	27.78 (±0.32)	2196.36 (±51.76)
December	305.00 (±73.18)	10.15 (±2.33)	85.55 (±18.27)	21.25 (±0.13)	24.88 (±0.20)	29.32 (±0.28)	2203.86 (±74.20)

Presentation of monthly data on cumulated and mean rainfall, minimum, maximum, and average temperature, and global solar radiation, 1 January 1998 to 31 December 2008 (Beaufonds-Miria station, Saint-Benoît).

The minimum monthly cumulated rainfall over the period from 1998 to 2008 was 17.6 mm (September 2001) and the maximum was 1,520.4 mm (February 1998). On average (± S.E.), the monthly mean temperature between 1998 and 2008 was 23.16±0.19°C and the average monthly global radiation was 1,806.11±30.63 J/cm^2^. December, January, February, and March were the wettest and warmest months, while October, August, and June were the driest months over the study period. July and August were the coolest months and the GSR was the highest (>2000 J/cm^2^) from October to January.

### Human cases of leptospirosis in Reunion Island between 1998 and 2008

**Figure 2:**
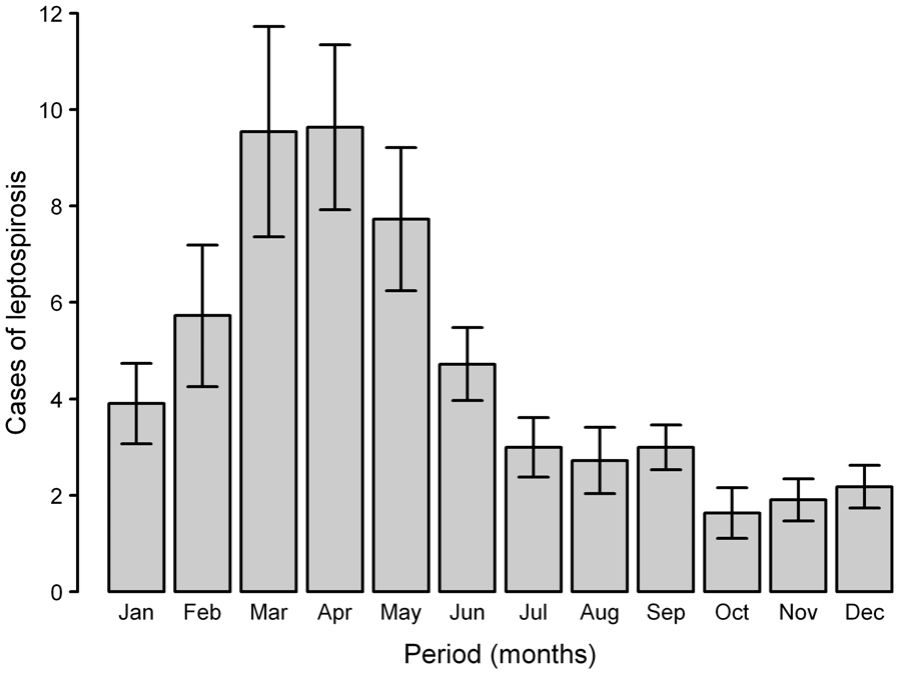
Monthly data on leptospirosis in Reunion Island. Mean number and standard error of monthly cases of leptospirosis in Reunion Island, 1 January 1998 to 31 December 2008.

There were 613 cases reported in Reunion Island between 1998 and 2008 (**Table 3**). The year with the highest number of cases was 2004, with 91 reported cases, while 1999 had the lowest number, 25 reported cases. The annual mean number of cases for this 11-year period was 55.73±6.61, and 359 cases (58.56%) were diagnosed between February and May (**Figure 2**). On average, the number of cases per month between 1998 and 2008 was 4.64±0.83. This number varied greatly over the period studied, from 0 to 27. The annual incidence of leptospirosis in Reunion Island ranged from 4.85 to 11.95 cases per 100,000 people between 1998 and 2008 (**Table 3**). The highest incidence was observed between 2003 and 2005, and then decreased by more than 50% to the end of the period.

**Table 3:** Annual and monthly cases of leptospirosis in Reunion Island.

	1998	1999	2000	2001	2002	2003	2004	2005	2006	2007	2008	Total	Mean of the monthly number of cases (± S.E.)
January	8	2	1	5	1	3	9	6	2	4	2	43	3.91 (±0.84)
February	**11**	3	6	4	6	4	**18**	3	2	5	1	63	5.73 (±1.47)
March	8	5	9	5	5	9	**20**	**27**	6	6	5	105	9.55 (±2.18)
April	3	5	6	9	7	**18**	**14**	**20**	**12**	8	4	106	9.64 (±1.71)
May	2	2	7	6	7	**19**	**10**	6	**12**	4	**10**	85	7.73 (±1.48)
June	9	4	2	2	3	8	4	8	5	4	3	52	4.73 (±0.75)
July	1	1	4	0	6	6	4	4	3	3	1	33	3.00 (±0.62)
August	2	0	1	2	4	8	2	4	4	0	3	30	2.73 (±0.69)
September	5	3	0	2	2	5	5	3	3	2	3	33	3.00 (±0.47)
October	2	0	0	2	5	3	0	0	3	3	0	18	1.64 (±0.53)
November	0	0	2	2	2	0	4	3	2	2	4	21	1.91 (±0.44)
December	1	0	3	4	3	1	1	2	5	2	2	24	2.18 (±0.44)
Total number of cases	52	25	41	43	51	84	91	86	59	43	38	613	55.73 (±6.61)
Mean of the monthly number of cases (±S.E.)	4.33 (±1.08)	2.08 (±0.56)	3.42 (±0.86)	3.58 (±0.70)	4.25 (±0.59)	7.0 (±1.75)	7.58 (±1.92)	7.17 (±2.32)	4.92 (±1.03)	3.58 (±0.61)	3.17 (±0.75)	/	/
Incidence (cases/100.000 inhab./year)	7.51	3.55	5.72	5.90	6.89	11.19	11.95	11.12	7.55	5.44	4.85		

In bold: monthly cases ≥10.

Presentation of the total number of cases of leptospirosis per month and per year in Reunion Island, mean monthly cases, and annual incidence (1 January 1998 to 31 December 2008).

**Figure 3:**
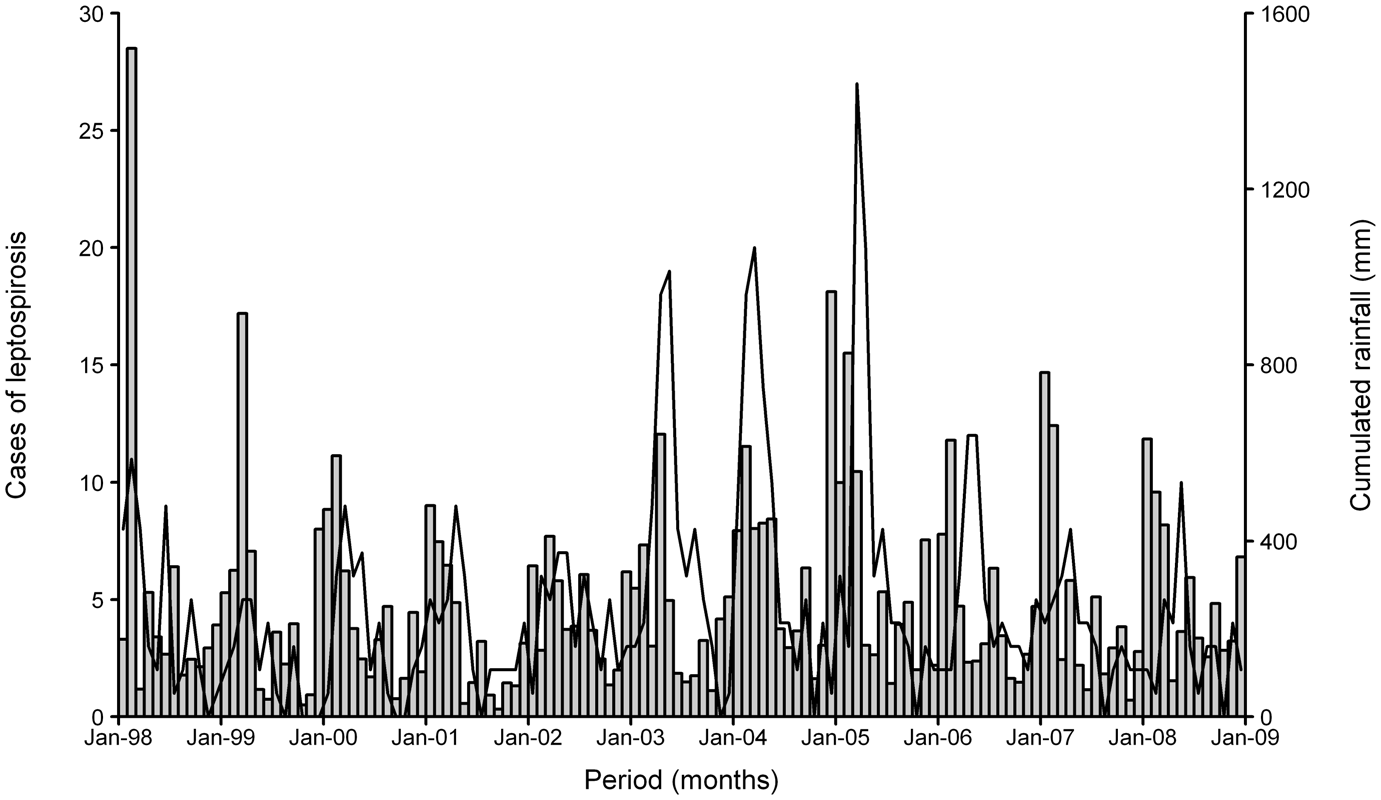
Leptospirosis and rainfall in Reunion Island. Monthly cases of leptospirosis (continuous line) and cumulated rainfall (bar chart), Beaufonds-Miria station, Saint-Benoît, 1 January 1998 to 31 December 2008.

The time-series of cases and the corresponding cumulated monthly rainfall are shown in **Figure 3**, while **Figures S1** and **S2** represent the time-series of cases and the corresponding average monthly temperature and GSR respectively.

Plots of ACF and PACF indicated a seasonal (annual) and cyclical pattern of diagnosis of cases of leptospirosis with an autoregressive order of less than 12 months. The time-series of cases appeared stationary after elimination of the calendar effects and removing of the seasonality (*p*-values of the augmented Dickey-Fuller tests were <0.05 for all corrected series).

Significant positive cross-correlations were detected between monthly cases of leptospirosis and monthly cumulated rainfall lagged by 0 and 2 months, and the strongest correlation (r = 0.28, *p*<0.01) was found with the average monthly rainfall recorded 2 months previously (**Table 4**).

**Table 4:** Cross-correlation between cases of leptospirosis and five meteorological parameters in Reunion Island.

	Lag (months)	0	1	2	3	4	5	6	7	8	9	10	11	12
Cumulated rainfall	Correlation	**0.2019** *	0.1525	**0.2825** *	0.1637	0.1076	0.0073	−0.0219	−0.1075	−0.0992	−0.1193	0.0499	0.1305	0.0954
	P-value	0.0202	0.0821	0.0011	0.0638	0.2265	0.9351	0.8078	0.2329	0.2732	0.1887	0.5852	0.1538	0.2998
Mean average temperature	Correlation	**0.1881** *	0.1668	**0.2267** *	0.1540	0.1661	0.0547	−0.0394	−0.0727	−0.1510	−0.1617	−0.0798	−0.019	0.1197
	P-value	0.0308	0.0569	0.0095	0.0815	0.0609	0.5415	0.6613	0.4201	0.0942	0.0740	0.3825	0.836	0.1927
Mean minimum temperature	Correlation	**0.2113** *	0.1865	**0.2629** *	0.1886	0.1913	0.0700	−0.0392	−0.0832	−0.1443	−0.1749	−0.0847	−0.0119	0.1166
	P-value	0.0150	0.0329	0.0025	0.0323	0.0305	0.4343	0.6630	0.3565	0.1100	0.0531	0.3536	0.8972	0.2049
Mean maximum temperature	Correlation	**0.1911** *	0.1680	**0.2080** *	0.1326	0.1277	0.0192	−0.0426	−0.0499	−0.1424	−0.1355	−0.0616	−0.0101	0.1305
	P-value	0.0281	0.0551	0.0175	0.1341	0.1509	0.8303	0.6361	0.5804	0.1146	0.1350	0.5005	0.9126	0.1553
Mean global solar radiation	Correlation	−0.0569	−0.0429	0.0822	0.0593	0.0916	0.1032	0.0163	−0.0016	−0.1532	**−0.2234** *	**−0.2153** *	−0.1704	0.0097
	P-value	0.5167	0.6265	0.3523	0.5041	0.3040	0.2482	0.8560	0.9855	0.0893	0.0130	0.0172	0.0616	0.9158

* Maximum of correlation between the monthly number of cases of leptospirosis and the meteorological factor considered.

Cross-correlation between 613 cases of leptospirosis reported in Reunion Island from 1 January 1998 to 31 December 2008, and cumulated monthly rainfall, mean monthly average temperature, mean monthly minimum temperature, mean monthly maximum temperature, and mean monthly solar global radiation, recorded in Saint-Benoît, lagged by 0–12 months.

Significant positive cross-correlations were detected between monthly cases of leptospirosis and average monthly temperature lagged by 0 and 2 months, and the strongest correlation (r = 0.23, *p*<0.01) was found with the average temperature recorded 2 months previously (**Table 4**).

Significant positive cross-correlations were detected between monthly cases of leptospirosis and monthly minimum temperature recorded 0, 1, 2, 3, and 4 months previously, while significant positive cross-correlations were detected between cases of leptospirosis and monthly maximum temperature recorded 0, 1, and 2 months previously (**Table 4**). Significant negative cross-correlations were detected between the number of cases of leptospirosis and average monthly GSR measured 9 and 10 months previously (**Table 4**). Nevertheless, correlations between GSR and the number of cases with a lag of 9 and 10 months are not biologically relevant, and consequently were not considered in the model. However, GSR measured 0 months prior to diagnosis was tested in the model to determine its relevance in the predictive model.

**Figure 4:**
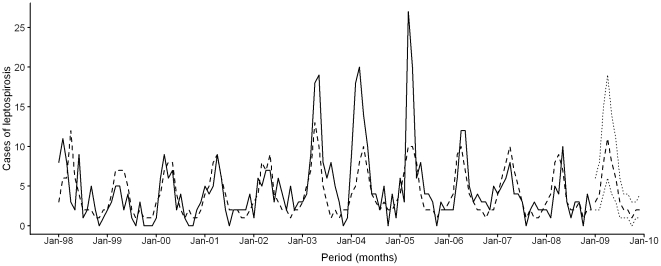
Observation and prediction of the monthly number of cases of leptospirosis in Reunion Island. Monthly number of cases reported by the CNRL from 1 January 1998 to 31 December 2008 (continuous line), number of cases predicted by the model from 1 January 1998 to 31 December 2009 (dotted line), and 95% confidence interval of the prediction for 2009 (dot-dashed line).

The best fitting ARIMAX model of cases of leptospirosis (*p* = 1, *d* = 0, *q* = 0; AIC = 208.29, estimated variance = 0.26, d.f. = 129) included the cases diagnosed in the previous month. Both the cumulated monthly rainfall and average monthly temperature recorded 0, 1, 2, and 3 months prior to diagnosis, as well as the average monthly GSR recorded 0 month prior to diagnosis were associated the model. Only the cumulated rainfall at a 2 months lag and the average temperature and GSR both at a 0 month lag were used in the model (**Table 5**). ACF and PACF of the residuals of the model appeared stationary (*not shown*) and the augmented Dickey-Fuller test confirmed the stationarity (*p* = 0.02). Between1998 and 2008, the correlation coefficient of our predictive model with the observed data is 0.677 (*p*<10^−4^) (**Figure 4**). With meteorological data from 2009, the ARIMAX model could make a prevision of the monthly number of leptospirosis cases with 95% confidence interval (**Figure 4** **,** **Table 6**).

**Table 5:** Best-fitting regression model of cases of leptospirosis in Reunion Island.

Variable	Lag (months)	*b*	S.E.	95% C.I.	*Student's t test*	*p-value*
Constant	-	3.397	2.984	[−2.452–9.246]	1.138	0.255
**Cases of leptospirosis**	**1**	**0.193**	**0.088**	**[0.02–0.366]**	**2.189**	**0.0286**
Cumulated rainfall	0	−0.007	0.079	[−0.162–0.148]	0.084	0.9327
Cumulated rainfall	1	0.027	0.069	[−0.108–0.163]	0.397	0.6913
**Cumulated rainfall**	**2**	**0.145**	**0.069**	**[0.011–0.28]**	**2.116**	**0.0343**
Cumulated rainfall	3	0.056	0.069	[−0.079–0.192]	0.813	0.4161
**Average temperature**	**0**	**3.836**	**1.445**	**[1.004–6.668]**	**2.655**	**0.0079**
Average temperature	1	−0.469	0.606	[−1.658–0.719]	0.774	0.4388
Average temperature	2	−0.482	0.625	[−1.706–0.742]	0.772	0.4401
Average temperature	3	−0.82	0.655	[−2.103–0.464]	1.252	0.2107
**Average global solar radiation**	**0**	**−1.293**	**0.494**	**[−2.26–−0.325]**	**2.618**	**0.0088**

*b*: coefficient value ; S.E.: standard error ; 95% C.I.: 95% confidence interval.

Parameters included in the best-fitting regression model of cases of leptospirosis (reported by the CNRL) in Reunion Island, 1 January 1998 to 31 December 2008 (AIC = 208.29, variance = 0.26, d.f: = 129).

**Table 6:** Predicted cases of leptospirosis in 2009, by month.

	Observed data	Prediction	95% C.I.
January 2009	5	3	2–6
February 2009	13	4	2–8
March 2009	9	8	4–15
April 2009	10	11	6–19
May 2009	6	8	4–14
June 2009	5	6	3–11
July 2009	4	3	1–6
August 2009	3	2	1–4
September 2009	5	2	1–4
October 2009	2	1	[0–3]
November 2009	3	2	1–3
December 2009	6	2	1–4
Total number of cases	71	52	

Presentation of the values predicted by the model for 2009 and 95% confidence interval (95% C.I.), compared to the data reported by the CNRL (the “observed data”) for the same year.

## Discussion

As in many developed countries, the incidence of leptospirosis in Reunion Island is decreasing: this is probably correlated to individual and collective prevention measures, a general increase in hygiene standards and the development of the tertiary sector. In contrast, recreational leptospirosis will probably become more common as water sports and nature activities increase in popularity. Efforts should be made to inform tourists about the disease. Rural leptospirosis may be an intractable problem given the complex ecological interactions involving domestic and wild reservoirs and environmental transmission sources. Results of the present study demonstrate that a combination of three meteorological parameters (rainfall, average temperature and GSR) could affect the number of human cases of leptospirosis. Our findings are largely consistent with hypotheses about the interactions of climatic factors in determining the strength and lag of weather effects on the incidence of leptospirosis. Certainly, our study shows that there is a link between monthly cases of leptospirosis in Reunion Island and both the cumulated rainfall recorded 2 months previously and average temperature and GSR, both recorded during the month considered. These three variables can be incorporated in a predictive model which can warn of future outbreaks of human leptospirosis in Reunion Island.

Only diagnosed forms (most of the time with hospitalization of the patient) are voluntarily reported to the CNRL (patients with a MAT titer ≥1/400, or with a positive PCR result, or with a positive blood culture). Consequently, the number of reported cases is significantly lower than the number of present cases of leptospirosis in Reunion Island [23]. A small number of cases could also be the result of poor epidemiological surveillance of the disease, leading to a decrease in the number of reported cases, and thus impacting on the validity of the data.

There are no comparable studies on Reunion Island providing an analysis over such a long time period as presented in this paper. Cases of leptospirosis were reported all year round, but epidemics showed seasonality since they mainly occurred during the rainy season, between February and May. Our results confirmed prior observations showing that the prevalence of the disease in Reunion Island is affected by rainfall [23], [26]. Therefore, despite the popular belief, the peak incidence of leptospirosis in Reunion Island was not during the sugar cane harvest (July to December) and other factors of contamination should be proposed. The prevalence on the west coast (annual rainfall inferior to 2,000 mm) was 0.712%. In areas with intermediate annual rainfall (2,000 to 4,000 mm), the prevalence was 1.444%, and in south-eastern areas (total annual rainfall superior to 4,000 mm), the prevalence was 3.093% [23]. Leptospirosis seasonality is also observed in French Polynesia where the monthly number of leptospirosis cases decreases during the dry season (July to October) [27] as well as in Martinique, where more than 35% of the annual cases occur during the rainy season (September to November).

The link between leptospirosis and rainfall is supported by previous observations made in Martinique, where a correlation was established between the occurrence of the disease and rainfall in the previous month [28] and in Guadeloupe, where the cycle of transmission begins about one month after the onset of heavy rain [29]. In Rio de Janeiro (Brazil), cluster case events between 1997 and 2002 were associated with heavy rainfall (OR 3.71; 95% CI 1.83–7.51) occuring 3–20 days before the onset of illness [30]. In Trinidad and Tobago, Mohan *et al.* [31] showed also a lag of 1–2 months between the onset of the rainy season (heavy rainfall) and the peak number of cases, and found a weak positive association between number of cases and amount of rainfall recorded each month (*r* = 0.56). The lag period of 1–2 months between heavy rainfall and cases is consistent with the probable effect of flooded land and water-soaked soils on leptospiral organism survival (1 to 2 months) and an average incubation period for leptospirosis of 1 to 3 weeks. In many parts of the world heavy rainfall and flooding can lead to outbreaks of leptospirosis, especially in tropical countries [19], [20] since transmission is often indirect in these areas.

During the rainy season the soil remains moist and can lead to the formation of pools of water which helps leptospires surviving for a longer period of time, and ultimately can lead to an increase in human and animal exposure to the bacteria. The model of Barcellos and Sabroza [32] shows that during dry periods, high leptospira concentrations in the soil are limited to a few metres around waste accumulation sources. During floods, the infectious bacteria can reach distant areas under the impact of water which increases the possibility of contact with all the population. In Reunion Island, as well as in many tropical countries, the frequency of flooding episodes may increase in the future due to changes in demographic patterns, destruction of ecologically sensitive areas such as wet lands, deforestation, reduction of the rural areas and climate change. Therefore the incidence of leptospirosis might increase in the future without the implementation of appropriate prevention strategies.

In our model, temperature was positively correlated with the case incidence at a lag of 0 months (*b* = 3.836). Variations of temperature are seasonal in Reunion Island, with high temperatures occurring during the rainy season and medium to cold temperatures occurring during the austral winter. Surface water temperatures follow the evolution of the air temperature, and in Reunion Island are highly dependent on geographical location and altitude. Thus survival of leptospires in water and soil may depend on the month, the nature of the water and location (east *vs* west, coast *vs* mountain).

Moreover, our results showed a negative correlation between the monthly cases and GSR recorded during the same month (*b* = −1.293). GSR is directly linked to the amount of UV radiation received at ground level and it is already known that UV exposure of more than two hours is lethal for leptospires [2]. GSR thus has an effect on the number of leptospirosis cases reported. Consequently, the dry season is clearly not favourable for leptospirosis transmission, since due to the low level of rainfall and high GSR (**Figures 3** **and S2**).

Our model, based on three meteorological parameters, estimates 67.7% of the variation of the monthly number of leptospirosis cases. Nevertheless, the peak incidence observed in February 2009 (13 cases) was not predicted by our model (number of cases predicted = 4), but the model gave a good prediction of the number of cases observed in March and April 2009 (observations were 9 and 10 cases respectively, while predictions were 8 and 11 respectively) (**Table 6**). Moreover, our model was not able to explain the major variations in the incidence of the disease observed in April and May 2003 (18 and 19 observed cases versus 10 and 13 predicted), March 2004 (20 observed cases versus 8 predicted), and March 2005 (27 observed cases versus 10 predicted). Thus, factors other than climatic must be taken into account to explain the highest monthly incidences of leptospirosis in Reunion Island.

In addition, the correlation between leptospirosis cases and the three meteorological parameters is weak (*b* = 0.145, *b* = 3.836, *b* = −1.293, for cumulated rainfall, mean temperature and solar global radiation respectively). In consequence, we can suppose that the model is not powerful enough to explain all the fluctuations in monthly cases, in particular when the number of cases is abnormally high. Our model can show the tendency of the incidence variations and, if it is undoubtedly a good predictor of monthly cases, it can not predict extreme values. Other parameters may greatly influence the survival of leptospires in the environment such as concentrations of oxygen and iron in water [2] or soil and water pH [33].

We did not include tropical cyclone data in our model because the difference between a depression, a tropical storm and a cyclone is mainly based on the wind speed which has a smaller impact than rainfall on the epidemiology of leptospirosis. Indeed, after cyclones, outbreaks of leptospirosis are mainly linked with flooding [34], [35].

Knowledge of the association between leptospirosis and three meteorological factors allows people to be alerted, particularly before the rainy season, of the risks present in the environment. This measure should achieve a reduction in exposure to leptospires during the high-risk period. Furthermore, as vaccinal immunity only lasts few months, our study shows that a seasonal vaccination based on the analysis of climatic data could be carried out on domestic animals. Individual measures of protection must be recommended particularly during the high-risk period, such as wearing gloves and boots when gardening, disinfecting the wound immediately in the case of a skin injury or avoiding drinking and submersion in flood or freshwater. Several serogroups are circulating in Reunion Island but the one mostly found in human cases is Icterohaemorrhagiae [23] followed by Canicola [23], [36]. Thus, rats and dogs are suspected to be the main carriers of leptospires on the island. They transmit the bacteria via infected urine, and the rainy season greatly favours the survival of the bacteria in the environment. Therefore, active rat control measures should be undertaken before and during the rainy season as well as the control of the stray dog populations and the cleaning of illegal dump sites.

## Supporting Information

Figure S1 **Leptospirosis and temperature in Reunion Island.** Monthly cases of leptospirosis (black curve) and average temperature (dotted curve). Beaufonds-Miria station, Saint-Benoît. 1 January 1998 to 31 December 2008.
(TIFF)

Figure S2 **Leptospirosis and global solar radiation in Reunion Island.** Monthly cases of leptospirosis (black curve) and global solar radiation (dotted curve). Beaufonds-Miria station, Saint-Benoît. 1 January 1998 to 31 December 2008.
(TIFF)

## References

[1] World Health Organization (1999). Leptospirosis worldwide, 1999.. Wkly Epidemiol Rec.

[2] Faine S, Adler B, Bolin C, Pérolat P (1999). *Leptospira* and leptospirosis.

[3] Katz AR, Ansdell VE, Effler PV, Middleton CR, Sasaki DM (2001). Assessment of the clinical presentation and treatment of 353 cases of laboratory-confirmed leptospirosis in Hawaii, 1974–998.. Clin Infect Dis.

[4] Levett PN (2001). Leptospirosis.. Clin Microbiol Rev.

[5] Monahan AM, Miller IS, Nally JE (2009). Leptospirosis: risks during recreational activities.. J Appl Microbiol.

[6] Priya CG, Hoogendijk KT, Berg MDV, Rathinam SR, Ahmed A (2007). Field rats form a major infection source of leptospirosis in and around Madurai, India.. J Postgrad Med.

[7] Tucunduva de Faria M, Calderwood MS, Athanazio DA, McBride AJA, Hartskeerl RA (2008). Carriage of Leptospira interrogans among domestic rats from an urban setting highly endemic for leptospirosis in Brazil.. Acta Trop.

[8] Brod CS, Aleixo JAG, Jouglard SDD, Fernandes CPH, Teixeira JLR (2005). Evidence of dog as a reservoir for human leptospirosis: a serovar isolation, molecular characterization and its use in a serological survey.. Rev Soc Bras Med Trop.

[9] Weekes CC, Everard COR, Levett PN (1997). Seroepidemiology of canine leptospirosis on the island of Barbados.. Vet Microbiol.

[10] Everard CO, Bennett S, Edwards CN, Nicholson GD, Hassel TA (1992). An investigation of some risk factors for severe leptospirosis on Barbados.. J Trop Med Hyg.

[11] Perrocheau A, Pérolat P (1997). Epidemiology of leptospirosis in New Caledonia (South Pacific): a one-year survey.. Eur J Epidemiol.

[12] Douglin CP, Jordan C, Rock R, Hurley A, Levett PN (1997). Risk factors for severe leptospirosis in the parish of St. Andrew, Barbados.. Emerg Infect Dis.

[13] Morgan J, Bornstein Shari L, Karpati Adam M, Bruce M, Bolin CA (2002). Outbreak of leptospirosis among triathlon participants and community residents in Springfield, Illinois, 1998.. Clin Infect Dis.

[14] Sejvar J, Bancroft E, Winthrop K, Bettinger J, Bajani M (2003). Leptospirosis in “eco-challenge” athletes, Malaysian Borneo, 2000.. Emerg Infect Dis.

[15] Picardeau M, Cornet M, Morel V, Sertour N, Chaumet D (2008). Impact of the revised diagnostic policy on the diagnosis and surveillance of leptospirosis in France [in French].. Bulletin Epidémiologique Hebdomadaire.

[16] Merien F, Portnoi D, Bourhy P, Charavay F, Berlioz-Athaud A (2005). A rapid and quantitative method for the detection of *Leptospira* species in human leptospirosis.. FEMS Microbiol Lett.

[17] Hermann Storck C, Postic D, Lamaury I, Perez JM (2008). Changes in epidemiology of leptospirosis in 2003–2004, a two El Nino southern oscillation period, Guadeloupe archipelago, French West Indies.. Epidemiol Infect.

[18] Sanders EJ, Rigau-Perez JG, Smits HL, Deseda CC, Vorndam VA (1999). Increase of leptospirosis in dengue-negative patients after a hurricane in Puerto Rico in 1996 [correction of 1966].. Am J Trop Med Hyg.

[19] Gaynor K, Katz AR, Park SY, Nakata M, Clark TA (2007). Leptospirosis on Oahu: an outbreak associated with flooding of a university campus.. Am J Trop Med Hyg.

[20] Pappachan MJ, Sheela M, Aravindan KP (2004). Relation of rainfall pattern and epidemic leptospirosis in the Indian state of Kerala.. J Epidemiol Community Health.

[21] Raunet M (1991). Physical environment and soils of Reunion. Consequences for effective agricultural and use [in French]; CIRAD-IRAT, editor.

[22] INSEE (Institut National de la Statistique et des Etudes Economiques) (2010). Tableau Economique de La Réunion (TER) Edition 2010..

[23] Duval G, Michault A, Baranton G, Law-Koune JD, Folio G (1991). Sero epidemiological survey on human leptospirosis in Reunion Island [in French].. Rev Epidémiol Santé Publique.

[24] Cryer JD, Kung-Sik C, Casella G, Fienberg S, Olkin I (2008). Time series analysis: With applications in R. Second edition;.

[25] Shumway RH, Stoffer DS (2006). Time series analysis and its applications: With R examples (Springer texts in statistics).

[26] Mailloux M, Debarbat F, Mollaret HH (1983). Leptospiroses in the island of Reunion. I. Human leptospiroses [in French].. Bull Soc Pathol Exot.

[27] Hirschauer C, Daudens E, Coudert C, Frogier E, Melix G (2009). Epidemiology of leptospirosis in French Polynesia from 2006 to 2008 [in French].. Bulletin Epidémiologique Hebdomadaire.

[28] Lhomme V, Grolier-Bois L, Jouannelle J, Elisabeth L (1996). Leptospirosis in Martinique from 1987 to 1992: results of an epidemiological, clinical and biological study.. Med Mal Infect.

[29] Herrmann-Storck C, Brioudes A, Quirin R, Deloumeaux J, Lamaury I (2005). Retrospective review of leptospirosis in Guadeloupe, French West Indies 1994–2001.. West Indian Med J.

[30] Tassinari WS, Pellegrini DCP, Sá CBP, Reis RB, Ko AI (2008). Detection and modelling of case clusters for urban leptospirosis.. Trop Med Int Health.

[31] Mohan ARM, Cumberbatch A, Adesiyun AA, Chadee DD (2009). Epidemiology of human leptospirosis in Trinidad and Tobago, 1996–2007: A retrospective study.. Acta Trop.

[32] Barcellos C, Sabroza PC (2001). The place behind the case: leptospirosis risks and associated environmental conditions in a flood-related outbreak in Rio de Janeiro.. Cad Saude Publica.

[33] Gordon Smith CE, Turner LH (1961). The effect of pH on the survival of leptospires in water.. Bull OMS.

[34] Sehgal SC, Sugunan AP, Vijayachari P (2002). Outbreak of leptospirosis after the cyclone in Orissa.. Natl Med J India.

[35] McCurry J (2009). Philippines struggles to recover from typhoons.. Lancet.

[36] Agésilas F, Gey F, Monbrunt A, Combes JC, Llanas B (2005). Acute leptospirosis in children in Reunion Island: a retrospective review of 16 cases [in French].. Arch Pediatr.

