# Supplementary figures and images for: Seasonality of Human Leptospirosis in Reunion Island (Indian Ocean) and Its Association with Meteorological Data
Source: PLoS One. 2011 May 31;6(5):e20377. doi: 10.1371/journal.pone.0020377 (PMC3105052; doi:10.1371/journal.pone.0020377)

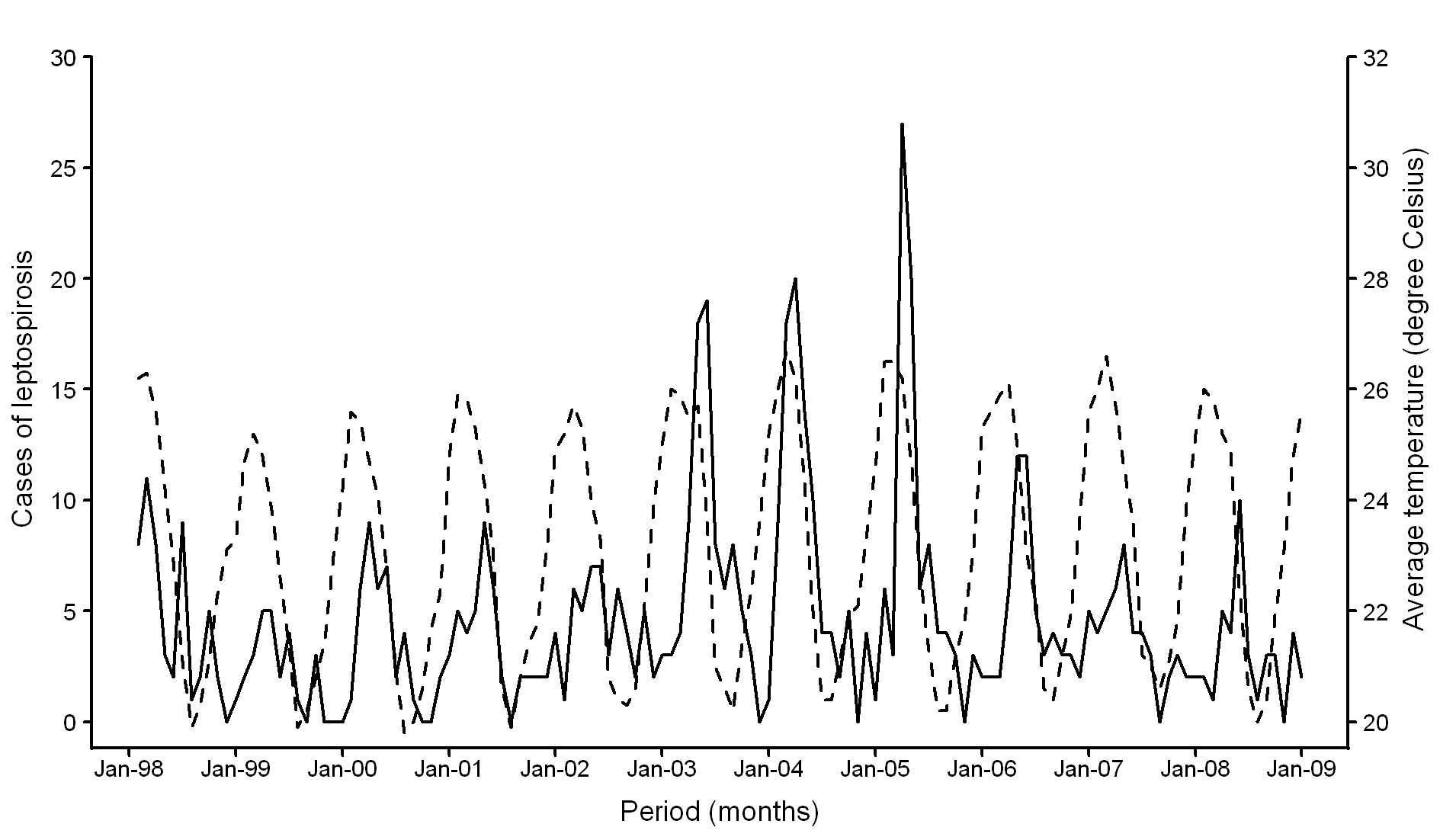

Supplement: Figure S1 — Leptospirosis and temperature in Reunion Island. Monthly cases of leptospirosis (black curve) and average temperature (dotted curve). Beaufonds-Miria station, Saint-Benoît. 1 January 1998 to 31 December 2008. (TIFF) [file pone.0020377.s001.tif]

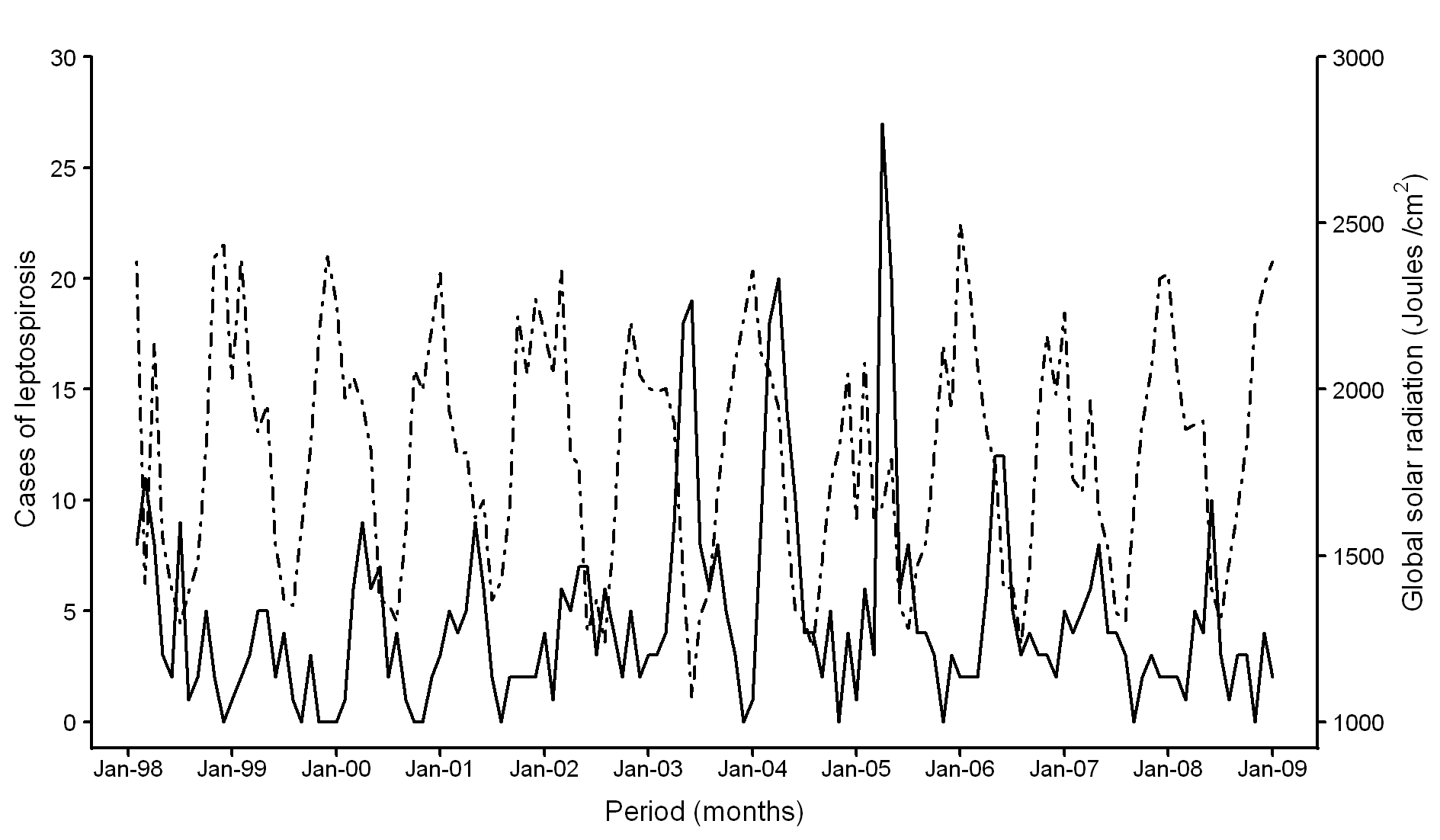

Supplement: Figure S2 — Leptospirosis and global solar radiation in Reunion Island. Monthly cases of leptospirosis (black curve) and global solar radiation (dotted curve). Beaufonds-Miria station, Saint-Benoît. 1 January 1998 to 31 December 2008. (TIFF) [file pone.0020377.s002.tif]
